# Non-Surgical Periodontal Therapy’s Influence on Alpha-Synuclein and Inflammatory Marker Levels: A Pilot Study

**DOI:** 10.3390/jcm13123586

**Published:** 2024-06-19

**Authors:** Patrícia Lyra, João Botelho, Silvia Rota, Karolina Poplawska-Domaszewicz, Vanessa Machado, Daniela Guerreiro, Luís Proença, Helena Barroso, José João Mendes, Kallol Ray Chaudhuri

**Affiliations:** 1 Egas Moniz Center for Interdisciplinary Research, Egas Moniz School of Health and Science, 2829-511 Almada, Portugal; plyra@egasmoniz.edu.pt (P.L.); jbotelho@egasmoniz.edu.pt (J.B.); vmachado@egasmoniz.edu.pt (V.M.); dguerreiro@egasmoniz.edu.pt (D.G.); lproenca@egasmoniz.edu.pt (L.P.); mhbarroso@egasmoniz.edu.pt (H.B.); 2 Department of Basic & Clinical Neuroscience, Institute of Psychiatry, Psychology & Neuroscience, King’s College London, London WC2R 2LS, UK; silvia.rota@kcl.ac.uk (S.R.); ray.chaudhuri@kcl.ac.uk (K.R.C.); 3 Parkinson’s Foundation Center of Excellence, King’s College Hospital, London SE5 9RS, UK; karolina.poplawska@usk.poznan.pl; 4 Department of Neurology, Poznan University of Medical Sciences, 60-355 Poznan, Poland

**Keywords:** alpha-synuclein, cytokines, periodontitis, periodontal disease, oral health, periodontal treatment, inflammatory burden

## Abstract

**Background**: Microbial dysbiosis may contribute to alpha-synuclein (α-Syn) homeostasis disruption, yet the burden of inflammatory periodontal infection and its treatment have never been studied in this regard. We aimed to compare the cytokine and α-Syn levels in the saliva and blood of patients with periodontitis who underwent non-surgical periodontal therapy (NSPT) and those of their healthy counterparts. **Methods**: Periodontal examination and saliva and blood sample collection were carried out in incoming patients at a university clinic. The periodontitis group (PG) received NSPT. The sample collection and periodontal observation were repeated 30 days after. IL-6, IL1-β and total α-Syn were quantified using immunoassay methods. The periodontal inflamed surface area (PISA) was calculated as a proxy for periodontal inflammation. **Results**: Eleven participants formed the PG, and there were fifteen healthy controls (HC). At baseline, no correlation between salivary and plasma α-Syn was found. The salivary α-Syn levels revealed a tendency to decrease 30 days after, particularly in the PD cases. The variation in PISA and α-Syn showed significant correlation. Salivary α-Syn correlated negatively with salivary IL-6 levels at both timepoints in the total sample (rho = −0.394 and rho = −0.451) and in the HC (rho = −0.632 and rho = −0.561). Variations in plasma IL-6 and α-Syn were negatively correlated (rho = −0.518) in the healthy participants. Baseline plasma IL1-β negatively correlated with plasmatic α-Syn at 30 days in the HC (rho = −0.581). **Conclusions**: Salivary and plasma α-Syn bioavailability operate independently, and periodontal diagnosis was not a confounding factor. Salivary α-Syn levels were significantly affected by NSPT, contrary to plasma levels. These results should be confirmed in future larger and prospective studies.

## 1. Introduction

Alpha-synuclein (α-Syn) is a short-sized protein involved in synaptic vesicle biology and neurotransmitter traffic [1], also found in blood and immune cells [2,3]. In α-Syn homeostasis disruption, toxic species form through protein aggregation into oligomers or protofibrils [4], spreading throughout the central nervous system (CNS), which might cause neurodegeneration [5,6]. Extracellularly, α-Syn can be found in body fluids or peripheral tissues, such as cerebrospinal fluid (CSF), saliva, serum, plasma, urine, tears, the salivary glands and various organs of the gastrointestinal tract [2,7,8,9,10,11]. Therefore, it has been reported as a promising biomarker in Parkinson’s disease (PD), the most common synucleinopathy [12]. The reason it accumulates is not fully known but is thought to be multifactorial, including aging, environmental influences (such as the dysbiosis of the gut commensal microflora), exposure to toxins and genetic mutations [13,14,15].

A significant source of inflammation is the globally prevalent periodontitis, an integral part of the gastrointestinal tract [16,17]. This condition affects the supporting tissues of the teeth due to the accumulation of dental biofilm, imbalanced microbiota and an immunological response [18]. If untreated, periodontal bacteria and their products spread and cause inflammation in extra-oral sites or the spillover of local inflammatory mediators from periodontal tissues into the bloodstream [19]. This triggers an inflammatory cascade, including high levels of IL-1, IL-6, C-reactive protein (CRP), TNF-α, fibrinogen and circulating neutrophils [19,20]. In addition to this low-grade inflammation, the resulting bacteremia is accompanied by toxic products, such as gingipains (from Porphyromonas gingivalis), which have been implicated in the pathology of several diseases, namely breast and colon cancer and rheumatoid arthritis, and in the brains of Alzheimer’s and PD patients [19,21,22,23].

We recently reviewed how microbial-dysbiosis-related inflammation may play a role in α-Syn pathways [3]. Mice exposed to lipopolysaccharide (LPS) exhibited α-Syn adopting a fibrillar form in the brain tissue, with cognitive deficits and enhanced dopaminergic neuronal loss, suggesting LPS-induced neuroinflammation and the periodontitis-related genetic background interact synergically [24,25]. Therefore, disruptions in commensal microbiota may be a driving force in α-synucleinopathies [15,26], as firmly established within the microbiota–gut–brain axis [26,27]. Gingipains, for example, have been shown to accumulate in dopaminergic neurons of the substantia nigra in humans, specifically interacting with α-Syn [28]. Nevertheless, research focuses on diagnosed neurodegeneration, but it is still unclear how the inflammatory burden of periodontal infection and its treatment impact synuclein homeostasis. This is relevant because it may unveil clues on pathogenesis and synergistic interactions, as α-Syn is a protein with physiological functions, novel insights into the gut–brain axis and even a role as a biomarker of periodontal health.

With this study, we aimed to assess the baseline salivary and blood levels of inflammatory markers of interest (IL-6 and IL1-β) and α-Syn in a case–control study. Additionally, we delivered non-surgical periodontal therapy (NSPT) in periodontitis patients and compared the salivary and blood surrogate variation at a 30-day follow-up with their healthy counterparts.

## 2. Materials and Methods

### 2.1. Study Setting, Eligibility Criteria and Sample Size

This study was a case–control study adjusted for sex at a 1:1 ratio. Incoming patients observed at the Egas Moniz Dental Clinic (EMDC) were included in consecutive sampling according to their periodontal status.

To be included, adult patients had to sign informed consent. The exclusion criteria were as follows: NSPT performed in the last year; antibiotic therapy, corticosteroids and immunosuppressive agents in the last 3 months; pregnant or lactating women; non-periodontal active inflammatory processes of the oral cavity.

The study was conducted in compliance with the relevant laws and institutional guidelines and was approved in January 2022 by the Egas Moniz Ethics Committee (Institutional Review Board, protocol 824). All the participants gave informed written consent to the study procedures, and the study was carried out in observance of the Declaration of Helsinki, as revised in 2013. This study is reported as per the STrengthening the Reporting of OBservational studies in Epidemiology (STROBE) guidelines (Supplementary Table S13) [29].

Considering the lack of studies comparing the impact of NSPT on α-Syn to provide a basis for sample size calculation, we conducted a preliminary pilot study with a sample size of 15 patients per group. This sample size was based on previous studies with a similar design [30,31].

### 2.2. Demographics Characteristics

Data were collected through a self-reported questionnaire on sociodemographic and behavioral aspects, including age, sex, educational level, marital status, smoking habits, oral care and hygiene-related behaviors (last dental visit, toothbrushing frequency, toothbrush type and interproximal cleaning) and diabetes mellitus (DM).

We categorically registered the education levels according to the 2011 International Standard Classification of Education (ISCED-2011) [32]: elementary (ISCED 1–2 levels), middle (ISCED 3–4 levels), higher (ISCED 5–8 levels). Marital status was categorized as Single, Married/Living with Partner or Divorced/Separated/Widowed. 

Smoking status was defined as non-smoker (category 0), former smoker (category 1) or active smoker (category 2), following a previous methodology [21]. Current smokers were those who had smoked ≥100 cigarettes during their lifetime and were still active smokers. Former smokers were defined as those who had smoked ≥100 cigarettes during their lifetime but had stopped. Those who had smoked <100 cigarettes during their lifetimes were categorized as never smokers.

DM cases were based on the patients’ auto-reports and then confirmed by medication regimens.

### 2.3. Periodontal Clinical Examination and Diagnosis

A full-mouth periodontal examination was performed at EMDC and during the 30-day follow-up consultations post-NSPT. All fully erupted teeth were examined using a manual periodontal North Carolina probe (Hu-Friedy; Chicago, IL, USA), and the number of missing teeth was recorded. Pocket depth (PD) was measured as the distance from the free gingival margin to the bottom of the pocket. Recessions (REC) represented the distance from the cementoenamel junction (CEJ) to the free gingival margin (a negative sign was assigned if the gingival margin was located coronally to the CEJ). Interdental clinical attachment loss (CAL) was calculated as the algebraic sum of the REC and PD measurements for each site. All the measurements were rounded to the lowest whole millimeter. Furcation involvement (FI) was assessed using a 2N probe (Hu-Friedy; Chicago, IL, USA), and tooth mobility was also appraised.

A periodontitis case implies interdental CAL ≥ 2 mm of non-adjacent teeth or buccal or oral CAL ≥ 3 mm, with PD > 3 mm detectable at ≥2 teeth [33]. The periodontitis staging was defined according to severity and extent. Regarding severity, it was categorized as mild (stage 1) if interdental CAL at the site of the greatest loss was 1–2 mm, moderate (stage 2) if it was 3–4 mm and severe (stage 3 and stage 4) if it was ≥5 mm. Additionally, the extent was described as localized (<30% of teeth involved), generalized (≥30% of teeth involved) or a molar/incisor pattern.

The periodontal inflamed surface area (PISA) was calculated as a proxy for periodontal inflammation. For each tooth, we computed the PISA as a continuous variable. The PISA comprised the surface area of the bleeding pocket epithelium [34,35].

### 2.4. Saliva Collection Protocol

Unstimulated saliva collection took place before a periodontal diagnosis at the EMDC and before clinical observation during the 30-day follow-up consultations post-NSPT. The minimum collection volume of unstimulated saliva was 5 mL [36].

Following saliva collection, the samples were stored at −80 °C. Upon analysis, after thawing, the samples were centrifuged at 9300× *g* for 5 min, and the supernatants were divided into 0.5 mL aliquots to be analyzed.

### 2.5. Blood Collection Protocol 

The blood sample collection took place before a periodontal diagnosis at the EMDC and before clinical observation during the 30-day follow-up consultations post-NSPT. Blood collection of 2.7 mL of venous blood was performed from the antecubital fossa using venipuncture, using a Safety-Multifly^®^ 21G needle with an 80 mm tube (SARSTEDT REF 85.1638.205) and Ethylenediaminetetraacetic acid-treated tubes (S-Monovette^®^ 2.7 mL K3E, 1.6 mg EDTA/mL) (SARSTEDT REF 05.1167.001) [7].

Following blood collection, the plasma samples underwent refrigerated centrifugation for 15 min at 1500× *g*. The resulting plasma supernatants were transferred into clean and low-residue polypropylene tubes. The samples were stored at −80 °C before examination.

### 2.6. Periodontal Treatment

Periodontitis cases underwent NSPT, performed in one or two sessions, according to the severity and extent of periodontitis. Subgingival instrumentation was performed through debridement, scaling and root planing. No adjuvant chemical therapy was prescribed. Patients requiring additional periodontal (surgical) treatment and re-evaluations were redirected to the periodontology department of the EMDC for continued treatment [37].

### 2.7. Salivary and Blood Circulating Markers 

Quantification of the salivary and blood levels of total α-synuclein was performed using the Human SNCα (Synuclein Alpha) ELISA Kit (REF E-EL-H0983-96T; Elabscience, Houston, TX, USA). As this study aims to investigate the physiological α-Syn levels in a population without a neurological diagnosis, we opted to quantify the total α-Syn levels, as oligomeric forms are mostly associated with PD and we did not want to discard the monomeric protein forms [10]. The levels of the inflammatory markers of interest (Il-1β and IL-6) were also measured by means of immunoassay kits—the High Sensitive ELISA Kit for Interleukin 6 (IL6) (REF HEA079Hu-96T; Cloud-Clone Corp., Houston, TX, USA) and the Human IL-1β (Interleukin 1 Beta) ELISA Kit (REF E-EL-H0149-96T; Elabscience, Houston, TX, USA). The assays were carried out in accordance with the manufacturer’s instructions.

### 2.8. Data Management and Statistical Analysis

Data analyses were performed using IBM SPSS Statistics v.29. The data were submitted for descriptive and inferential analyses. The association between categorical variables was checked using appropriate inferential statistical methodologies (chi-squared and Fisher’s exact test). Inferential comparison of the biomarker levels between groups was performed using Student’s *t*-test (data’s adequacy to normality was previously checked). The correlation between the inflammatory marker levels was assessed using Spearman’s rank correlation coefficient. A 5% significance level (*p* ≤ 0.05) was established in the inferential analyses. 

## 3. Results

### 3.1. Participants/Sample Description/Population

**Figure 1:**
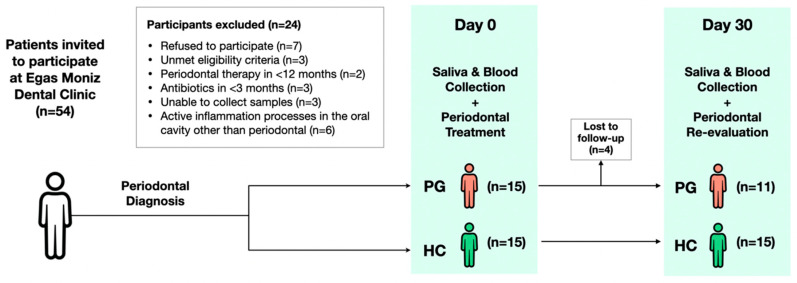
Flowchart of participants. Abbreviations: n, number of participants; PG, periodontitis group; HC, healthy controls.

A total of 54 new patients at the EMDC were invited to participate in the study (Figure 1), and 24 participants were excluded either because they refused to participate or did not meet the eligibility criteria or due to difficulties in sample collection. Thus, 30 participants were initially enrolled; however, 4 patients were lost mid-study and did not complete the programmed follow-up consultation. After clinical periodontal diagnosis, 11 patients formed the PG, while 15 formed the HC. Within the PG, there were five cases of stage II periodontitis (two of grade A and three of grade B), five cases of stage III periodontitis (two of grade A and three of grade B) and one case of stage IV periodontitis (grade C).

The participants’ sociodemographic and oral health behavior data are detailed in Table 1. The final sample presented a majority of women (53.8%). The periodontitis participants were significantly older than their healthy counterparts (*p* = 0.024). Most of the participants reported a middle educational level (57.7%) and being married/living with a partner (46.2%), and half reported non-smoking habits (50.0%). Regarding oral care habits, 61.5% of the participants reported their last dental visit within the last six months, brushing their teeth twice or more a day (96.2%) and mostly using a manual toothbrush (84.6%). The interproximal cleaning habits significantly differed between the case group and the HC, with the PG having a lower proportion of reported interproximal hygiene habits (*p* = 0.005). Only three patients (11.5% from total) reported a diagnosis of diabetes mellitus.

### 3.2. Plasma and Saliva Levels before and after Treatment

**Figure 2:**
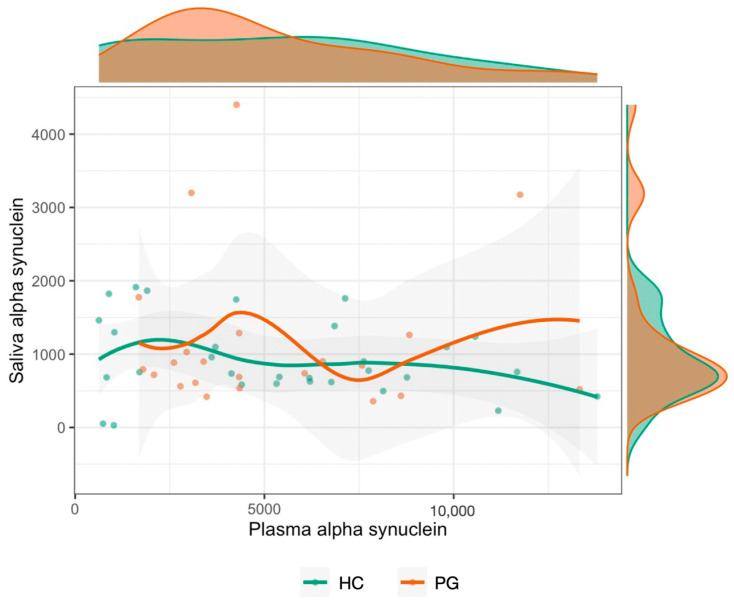
Relationship between baseline blood and salivary a-synuclein levels with respective distribution. The flattened, almost linear plot inside the graph seems to indicate no relationship between saliva and plasma α-Syn levels at baseline. Outside the plot, the distribution of baseline α-Syn plasma levels in periodontal cases is mostly concentrated to the left (top section), and baseline salivary levels are mostly concentrated to the right (lateral section). Abbreviations: PG, periodontitis group; HC, healthy controls.

**Figure 3:**
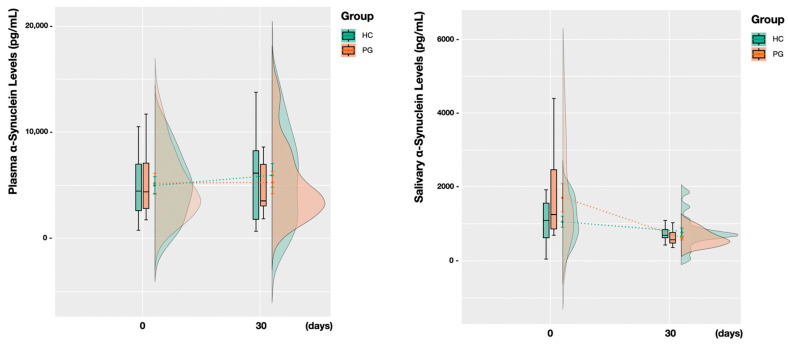
Plasma and salivary α-synuclein levels at baseline and follow-up. Regarding plasma α-Syn levels, both the average values and distribution of values at baseline and at the 30-day follow-up remained very similar between groups. However, salivary α-Syn levels revealed a decrease in mean levels (closer to the HC) and a shortening in the distribution of values. Abbreviations: PG, periodontitis group; HC, healthy controls.

**Figure 4:**
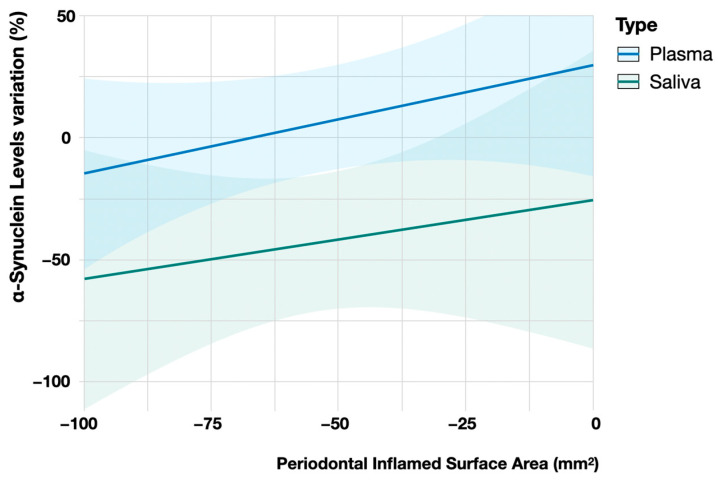
Salivary and plasma alpha-synuclein variation vs. periodontal inflamed surface area variation after periodontal therapy. Comparing baseline with follow-up values, we observed a higher negative α-Syn variation in saliva than in plasma (Figure 3). The higher the reduction in periodontal inflamed surface area, the more likely finding a higher negative variation in α-synuclein levels compared to the baseline was (as was previously supported in Figure 3). When the reduction in PISA was complete (that is, equal to −100%), a greater effect on α-Syn was noticed.

Our results seem to indicate no dependent association between the saliva and plasma α-Syn levels at baseline (Figure 2). Furthermore, throughout the time course of the study, no significant variations were seen in plasma α-Syn levels in either group (Figure 3). However, salivary α-Syn levels revealed a decrease by the 30-day mark, especially in the PG, not only in the mean levels but also in a shortening in the distribution of values (Figure 3). Accordingly, following periodontal treatment, the higher the reduction in the periodontal inflamed surface area (PISA), the more likely a higher negative variation in α-Syn levels was compared to the baseline (Figure 4).

We found no differences in the α-Syn levels in either group at baseline or follow-up for either saliva or blood (Table 2). Regarding the IL-6 analysis, no differences were found in the mean salivary levels of IL-6 between groups at baseline or follow-up. However, significant differences between the PG and HC were found regarding the IL-6 plasma levels, indicating that the PG presented higher mean IL-6 values at baseline (*p* = 0.008) and follow-up (*p* = 0.034). When analyzing the time-dependent behavior (from T0 to T30), a significant difference was found for the IL1-β salivary level mean difference (*p* = 0.012) when comparing both groups. This was reflected by an increase in the PG and a decrease for the HC.

### 3.3. Correlation Analysis of Markers

Correlations between the salivary and plasma markers both in the overall sample and according to periodontal status are detailed in the Supplementary File. Salivary α-Syn showed a significant negative correlation with salivary IL-6 in the overall sample (rho = −0.394 and rho = −0.451) and in the HC (rho = −0.632 and rho = −0.561), both at baseline and 30 days after NSPT (Supplementary Tables S1, S2, S7 and S8). Furthermore, salivary IL-6 showed a significant negative correlation with salivary IL1-β in the overall sample (rho = −0.755 and rho = −0.535) and in both groups—HC (rho = −0.718 and rho = −0.518) and PG (rho = −0.664 and rho = −0.818)—at baseline and 30 days after NSPT, respectively (Supplementary Tables S1, S2, S4, S5, S7 and S8). There was also a significant negative correlation between IL-6 plasma levels and IL1-β plasma levels (rho = −0.643) and α-Syn plasma levels (rho = −0.518) in the HC (Supplementary Table S9). When analyzing the marker levels at baseline versus at 30-day follow-up, our results show a significant negative correlation between salivary α-Syn at baseline and salivary IL-6 at 30-day follow-up (rho = −0.492) and vice versa (rho = −0.562 **) in the total sample (Supplementary Table S10), as well as in the HC (rho = −0.729 and rho = −0.657) (Supplementary Table S12). In addition, salivary IL-6 at baseline significantly negative correlates with salivary IL1-β at the 30-day follow-up in the overall sample (rho = −0.478 and rho = −0.675) and in both the PG (rho = −0.664 and rho = −0.764) and the HC (rho = −0.518 and rho = −0.532) (Supplementary Tables S10–S12). Finally, plasmatic IL1-β at baseline shows a significant negative correlation with plasmatic α-Syn at 30-day follow-up in the HC (rho = −0.581) (Supplementary Table S12).

## 4. Discussion

The salivary and blood levels of α-Syn and inflammatory markers of interest (IL-6 and IL1-β) were assessed according to periodontal status at baseline and 30 days following NSPT for periodontitis cases. To the best of our knowledge, this is the first time the monthly variation in α-Syn levels has been described in a group of patients with periodontitis. To date, there has been no previous mention of the monthly variation in α-Syn in a population without any kind of neurological diagnosis, and therefore this is also a novel report regarding the HC.

Our results show no linear association between the saliva and plasma α-Syn levels at baseline (Figure 2), indicating that the bioavailability of both markers seems to function independently. Also, the similar behavioral tendency of both groups at baseline seems to indicate that a periodontal diagnosis does not influence the overall variations in the α-Syn plasma and salivary levels.

When it comes to analyzing the effects of NSPT on α-Syn levels, our results show that throughout the time course of the study, no significant variations were seen in the plasma α-Syn levels (Figure 3). However, the salivary α-Syn levels revealed a decrease by the 30-day mark, especially in the PG, not only in the mean levels but also in a shortening in the distribution of values (Figure 4). In other words, comparing the baseline with the follow-up values, we observed a higher negative α-Syn variation in the saliva than in the plasma after periodontal treatment. In fact, the higher the reduction in the PISA, the more likely we were to find a higher negative variation in the α-Syn levels compared to the baseline (Figure 4). Ultimately, when the reduction in the PISA was complete, a greater effect on salivary α-Syn was noticed.

Therefore, NSPT in the periodontitis cases seem to be a confounding factor in the evaluation of α-Syn salivary levels. This shows the clinical implications of our results, which present NSPT as a confounding factor in the medical diagnosis using biomarkers, specifically salivary α-Syn. In fact, recent evidence points to salivary α-Syn as a diagnostic biomarker in α-synucleinopathies, and this is a subject of increasing interest nowadays [8,9,10]. These results indicate that recent NSPT seems to have a local effect in the oral cavity and must be taken into consideration by health professionals when analyzing α-Syn salivary levels for diagnosis purposes. NSPT with scaling may possibly lead to some local inflammation, which may have implications for salivary α-Syn values.

The characteristics of our periodontitis group align with the literature, as the periodontitis patients were significantly older than their healthy counterparts (*p* = 0.024) and performed lower levels of interproximal hygiene (*p* = 0.005) [38,39]. Also, the periodontitis group presented significantly higher baseline (*p* = 0.008) and follow-up (*p* = 0.034) IL-6 plasma values, corroborating with numerous studies reporting that markers of inflammatory response are elevated in subjects suffering from periodontitis, as periodontitis is an inflammatory disease that awakes the immune response with a cascade of inflammatory markers that are not limited to the diseased sites [40]. Furthermore, a significant difference was found for the IL1-β salivary level mean difference (*p* = 0.012) when comparing the overtime variation (from T0 to T30) in both groups. Our results showed an increase in the PG and a decrease in the HC. The literature supports that when analyzing a large number of blood inflammatory markers in response to periodontal treatment, great heterogeneity regarding the systemic response and inflammatory markers behavior has been reported [41]. Also, a recent study analyzing blood samples reported a variation in the number of peaks after periodontal treatment, with some of them increasing immediately after 1 month and decreasing again at 3 months and vice versa [42]. However, when it comes to saliva samples, a previous meta-analysis showed no statistically significant decrease or alterations in the levels of IL-1β and other inflammatory markers after NSPT between treatment and healthy control groups [43], suggesting the need for cautious interpretation of the present results.

The small size of our sample is the main limitation of this study, and therefore the results should be interpreted with caution, as there are inherent biases in case–control studies. The correlations between salivary and plasma markers according to periodontal status must also be considered carefully due to the small sample size, although it reveals clues to be clarified in future studies. Also, other systemic inflammatory diseases may have been present at the same time. However, this case–control study had a 10-month inclusion period and followed the strict STROBE guidelines [29], as detailed in Supplementary Table S13. Although this is not an ecological study per se, it observes the normal behavior of a group of people, and even the clinical intervention is a routine treatment for periodontitis.

With the increase in life expectancy over recent decades and the growing number of patients with synucleinopathies [12], it is crucial to gain a better understanding of the behavior of α-Syn and other inflammatory markers to develop novel clinical strategies to alter disease progression or even prevent disease onset.

## 5. Conclusions

Salivary and plasma α-Syn levels showed no association with periodontal diagnosis. NSPT seems to affect salivary α-Syn levels but not the plasma. NSPT may be a confounding factor when using salivary α-Syn as a biomarker. Future research should confirm these preliminary data using larger and longer prospective studies.

## Figures and Tables

**Table 1:** Participants’ sociodemographic and oral health behavioral data.

Variable	Total (*n* = 26)	HC (*n* = 15)	PG (*n* = 11)	*p*-Value *
Age, mean (SD) (Min–Max)	50.1 (17.8) (19–77)	43.5 (18.6) (19–70)	59.1 (12.3) (40–77)	0.024 *
Sex, % (*n*)				
Female	53.8 (14)	46.7 (7)	63.6 (7)	0.391
Male	46.2 (12)	53.3 (8)	36.4 (4)	
Educational Level, % (*n*)				
Elementary	15.4 (4)	13.3 (2)	18.2 (2)	-
Middle	57.7 (15)	60.0 (9)	54.5 (6)	
Higher	26.9 (7)	26.7 (4)	27.3 (3)	
Marital Status, % (*n*)				
Single	34.6 (9)	40.0 (6)	27.3 (3)	-
Married/Living with Partner	46.2 (12)	53.3 (8)	36.4 (4)	
Divorced/Separated/Widowed	19.2 (5)	6.7 (1)	36.4 (4)	
Smoking Habits, % (*n*)				
Non-Smoker	50.0 (13)	53.3 (8)	45.5 (5)	-
Former Smoker	26.9 (7)	26.7 (4)	27.3 (3)	
Active Smoker	23.1 (6)	20.0 (3)	27.3 (3)	
Last Dental Visit, % (*n*)				
<6 Months	61.5 (16)	66.7 (10)	54.5 (6)	-
6–12 Months	15.4 (4)	13.3 (2)	18.2 (2)	
>12 Months	23.1 (6)	20.0 (3)	27.3 (3)	
Toothbrushing, % (*n*)				
Once a Day	3.8 (1)	0.0 (0)	9.1 (1)	-
Twice or More a Day	96.2 (25)	100.0 (15)	90.9 (10)	
Toothbrush Type, % (*n*)				
Manual	84.6 (22)	93.3 (14)	72.7 (8)	0.279
Electric	15.4 (4)	6.7 (1)	27.3 (3)	
Interproximal Cleaning, % (n)				
No	76.9 (20)	73.3 (11)	81.8 (9)	0.005 *
Often/Yes	23.1 (6)	26.7 (4)	18.2 (2)	
Diabetes Mellitus, % (*n*)				
Yes	11.5 (3)	0.0 (0)	27.3 (3)	-
No	88.5 (23)	100.0 (15)	72.7 (8)	

* Chi-square/Fisher’s exact test, except for “age”, where Student’s *t*-test was used. Significant *p*-values (*p* < 0.05) denoted with an asterisk (*). Abbreviations: SD, standard deviation; *n*, number of participants; Min, minimum; Max, maximum.

**Table 2:** Biomarker levels (plasma and salivary) before (T0) and after treatment (T30).

Variable	Total (*n* = 26)	HC (*n* = 15)	PG (*n* = 11)	*p*-Value *
**α-synuclein (pg/mL), mean (SD)**				
Salivary, T0	1365.1 (964.4)	1090.4 (566.3)	1739.6 (1268.3)	0.090
Plasma, T0	5067.2 (3091.9)	4977.9 (3155.6)	5189.1 (3151.1)	0.867
Salivary, T30	711.0 (369.6)	772.9 (449.3)	626.7 (212.9)	0.329
Plasma, T30	5643.1 (3883.3)	5929.5 (4267.5)	5252.6 (3451.5)	0.670
Salivary, (Diff., T30-T0)	−654.0 (1021.6)	−317.5 (452.1)	−1112.8 (1386.3)	0.093
Plasma, (Diff., T30-T0)	575.9 (1948.8)	951.6 (1844.3)	63.6 (2057.3)	0.259
**IL-6 (pg/mL), mean (SD)**				
Salivary, T0	6.0 (7.8)	4.4 (6.2)	8.1 (9.4)	0.240
Plasma, T0	6.3 (3.7)	4.7 (3.8)	8.4 (2.2)	0.008 *
Salivary, T30	6.4 (7.8)	6.2 (9.2)	6.6 (5.6)	0.908
Plasma, T30	4.7 (3.0)	3.6 (3.0)	6.1 (2.5)	0.034 *
Salivary, (Diff., T30-T0)	0.4 (7.2)	1.8 (8.4)	−1.5 (5.1)	0.256
Plasma, (Diff., T30-T0)	−1.6 (2.0)	−1.1 (1.7)	−2.3 (2.4)	0.137
**IL1-β (pg/mL), mean (SD)**				
Salivary, T0	462.3 (318.4)	502.2 (358.4)	407.8 (260.7)	0.466
Plasma, T0	162.6 (80.5)	151.2 (89.5)	178.0 (67.2)	0.412
Salivary, T30	468.0 (385.4)	421.3 (398.6)	531.6 (375.8)	0.482
Plasma, T30	84.4 (52.0)	77.6 (45.9)	93.7 (60.3)	0.447
Salivary, (Diff., T30-T0)	−5.7 (212.1)	−80.9 (169.3)	123.8 (213.8)	0.012 *
Plasma, (Diff., T30-T0)	−78.2 (52.3)	−73.6 (51.3)	−84.4 (55.7)	0.614

* Student’s *t*-test. Significant *p*-values (*p* < 0.05) denoted with an asterisk (*). Abbreviations: SD, standard deviation; *n*, number of participants; Diff, difference; T0, baseline; T30, follow-up; α-Syn, alpha-synuclein; IL-6, interleukin 6; IL1-β, interleukin 1-β.

## Data Availability

The original contributions presented in the study are included in the article/supplementary material; further inquiries can be directed to the corresponding author.

## Supplementary Materials

The following supporting information can be downloaded at https://www.mdpi.com/article/10.3390/jcm13123586/s1. Table S1. Correlation between biomarker levels of the overall sample at baseline (T0); Table S2. Correlation between biomarker levels of the overall sample at the 30-day follow-up (T30); Table S3. Correlation between the variation (Δ) in biomarker levels of the overall sample (T30-T0); Table S4. Correlation between biomarker levels of the periodontitis group at baseline (T0); Table S5. Correlation between biomarker levels of the periodontitis group at 30-day follow-up (T30); Table S6. Correlation between the variation (Δ) in biomarker levels of the periodontitis group (T30-T0); Table S7. Correlation between biomarker levels of the healthy control group at baseline (T0); Table S8. Correlation between biomarker levels of the healthy control group at 30-day follow-up (T30); Table S9. Correlation between the variation (Δ) in biomarker levels of the healthy control group (T30-T0); Table S10. Correlation between biomarker levels of the overall sample at baseline (T0) and at 30-day follow-up (T30); Table S11. Correlation between biomarker levels of the periodontitis group at baseline (T0) and at 30-day follow-up (T30); Table S12. Correlation between biomarker levels of the control group at baseline (T0) and at 30-day follow-up (T30); Table S13. STROBE statement—checklist of items that should be included in reports of case–control studies.
