# Supplementary material for: Non-Surgical Periodontal Therapy’s Influence on Alpha-Synuclein and Inflammatory Marker Levels: A Pilot Study
Source: J Clin Med. 2024 Jun 19;13(12):3586. doi: 10.3390/jcm13123586 (PMC11204787; doi:10.3390/jcm13123586)
Supplement: Supplementary file 1 [file jcm-13-03586-s001.zip › jcm-3046874-supplementary.pdf]

Supplementary Table S1. Correlation between biomarker levels of the overall sample at baseline (T0)

**Overall (N=26)**						
**T0**	α-Syn, plasma	α-Syn, saliva	IL-6, plasma	IL-6, saliva	IL1-β, plasma	IL1-β, saliva
α-Syn, plasma	-	-.085	.022	.084	-.203	-.104
α-Syn, saliva	-	-	-.110	**-.394**	**.704**	.336
IL-6, plasma	-	-	-	**.389**	.039	-.041
IL-6, saliva	-	-	-	-	-.384	**-.755**
IL1-β, plasma	-	-	-	-	-	.374
IL1-β, saliva	-	-	-	-	-	-

* Spearman's correlation coefficient (rho). Significant ($p<0.05$) rho values denoted in bold. Abbreviations: n, number of participants; T0, baseline; α-Syn, alpha-synuclein; IL-6, interleukin 6; IL1-β, interleukin 1-β.

Supplementary Table S2. Correlation between biomarker levels of the overall sample at the 30-days follow-up (T30)

**Overall (N=26)**						
**T30**	α-Syn, plasma	α-Syn, saliva	IL-6, plasma	IL-6, saliva	IL1-β, plasma	IL1-β, saliva
α-Syn, plasma	-	-.352	.006	.209	-.050	-.007
α-Syn, saliva	-	-	-.330	**-.451**	-.077	.175
IL-6, plasma	-	-	-	.055	.382	**.550**
IL-6, saliva	-	-	-	-	-.385	**-.535**
IL1-β, plasma	-	-	-	-	-	.253
IL1-β, saliva	-	-	-	-	-	-

* Spearman's correlation coefficient (rho). Significant ($p<0.05$) rho values denoted in bold. Abbreviations: n, number of participants; T30, follow-up; α-Syn, alpha-synuclein; IL-6, interleukin 6; IL1-β, interleukin 1-β.

Supplementary Table S3. Correlation between the variation (Δ) of biomarker levels of the overall sample (T30-T0)

**Overall (N=26)**						
**Δ (T30-T0)**	α-Syn, plasma	α-Syn, saliva	IL-6, plasma	IL-6, saliva	IL1-β, plasma	IL1-β, saliva
α-Syn, plasma	-	.150	-	-	-	-
α-Syn, saliva	-	-	-	-	-	-
IL-6, plasma	-.300	**-.438**	-	-.102	-	-
IL-6, saliva	**.441**	.071	-	-	-	-
IL1-β, plasma	.195	.056	-.185	.013	-.225	-
IL1-β, saliva	.134	-.231	-.147	-.182	-	-

* Spearman's correlation coefficient (rho). Significant (p<0.05) rho values denoted in bold. Abbreviations: n, number of participants; Δ, variation between baseline and follow-up; T0, baseline; T30, follow-up; α-Syn, alpha-synuclein; IL-6, interleukin 6; IL1-β, interleukin 1-β.

Supplementary Table S4. Correlation between biomarker levels of the periodontitis group at baseline (T0)

**Periodontitis Group (n=11)**						
**T0**	α-Syn, plasma	α-Syn, saliva	IL-6, plasma	IL-6, saliva	IL1-β, plasma	IL1-β, saliva
α-Syn, plasma	-	.036	-.064	.282	.155	-.055
α-Syn, saliva	-	-	-.318	.009	.427	-.127
IL-6, plasma	-	-	-	-.336	.300	.427
IL-6, saliva	-	-	-	-	-.018	**-.664**
IL1-β, plasma	-	-	-	-	-	.136
IL1-β, saliva	-	-	-	-	-	-

* Spearman's correlation coefficient (rho). Significant (p<0.05) rho values denoted in bold. Abbreviations: n, number of participants; T0, baseline; α-Syn, alpha-synuclein; IL-6, interleukin 6; IL1-β, interleukin 1-β.

Supplementary Table S5. Correlation between biomarker levels of the periodontitis group at the 30-days follow-up (T30)

**Periodontitis Group (n=11)**						
**T30**	α-Syn, plasma	α-Syn, saliva	IL-6, plasma	IL-6, saliva	IL1-β, plasma	IL1-β, saliva
α-Syn, plasma	-	**-.636**	.210	.264	.455	-.291
α-Syn, saliva	-	-	-.310	-.336	**-.727**	.409
IL-6, plasma	-	-	-	-.515	.460	**.715**
IL-6, saliva	-	-	-	-	.027	**-.818**
IL1-β, plasma	-	-	-	-	-	-.145
IL1-β, saliva	-	-	-	-	-	-

* Spearman's correlation coefficient (rho). Significant (p<0.05) rho values denoted in bold. Abbreviations: n, number of participants; T30, follow-up; α-Syn, alpha-synuclein; IL-6, interleukin 6; IL1-β, interleukin 1-β.

Supplementary Table S6. Correlation between the variation (Δ) of biomarker levels of the periodontitis group (T30-T0)

**Periodontitis Group (n=11)**						
**Δ (T30-T0)**	α-Syn, plasma	α-Syn, saliva	IL-6, plasma	IL-6, saliva	IL1-β, plasma	IL1-β, saliva
α-Syn, plasma	-	.227	-	-	-	
α-Syn, saliva	-	-	-	-	-	
IL-6, plasma	-.236	**-.900**	-	-.155	-	
IL-6, saliva	**.764**	.064	-	-	-	

IL1-β, plasma	-.182	-.145	.236	-.291	-	**-.664**
IL1-β, saliva	-.255	-.318	.364	-.182	-	-

* Spearman's correlation coefficient (rho). Significant (p<0.05) rho values denoted in bold. Abbreviations: n, number of participants; Δ, variation between baseline and follow-up; T0, baseline; T30, follow-up; α-Syn, alpha-synuclein; IL-6, interleukin 6; IL1-β, interleukin 1-β.

Supplementary Table S7. Correlation between biomarker levels of the healthy controls group at baseline (T0)

**Control Group (n=15)**						
**T0**	α-Syn, plasma	α-Syn, saliva	IL-6, plasma	IL-6, saliva	IL1-β, plasma	IL1-β, saliva
α-Syn, plasma	-	-.093	.064	-.021	-.466	.189
α-Syn, saliva	-	-	**-.539**	**-.632**	**.856**	.507
IL-6, plasma	-	-	-	**.596**	-.388	-.321
IL-6, saliva	-	-	-	-	**-.538**	**-.718**
IL1-β, plasma	-	-	-	-	-	.436
IL1-β, saliva	-	-	-	-	-	-

* Spearman's correlation coefficient (rho). Significant (p<0.05) rho values denoted in bold. Abbreviations: n, number of participants; T0, baseline; α-Syn, alpha-synuclein; IL-6, interleukin 6; IL1-β, interleukin 1-β.

Supplementary Table S8. Correlation between biomarker levels of the healthy controls group at the 30-days follow-up (T30)

**Control Group (n=15)**						
**T30**	α-Syn, plasma	α-Syn, saliva	IL-6, plasma	IL-6, saliva	IL1-β, plasma	IL1-β, saliva
α-Syn, plasma	-	-.264	-.029	.275	-.443	.146
α-Syn, saliva	-	-	-.236	**-.561**	.361	.093
IL-6, plasma	-	-	-	-.011	.361	.507
IL-6, saliva	-	-	-	-	**-.654**	**-.518**
IL1-β, plasma	-	-	-	-	-	**.579**
IL1-β, saliva	-	-	-	-	-	-

* Spearman's correlation coefficient (rho). Significant (p<0.05) rho values denoted in bold. Abbreviations: n, number of participants; T30, follow-up; α-Syn, alpha-synuclein; IL-6, interleukin 6; IL1-β, interleukin 1-β.

Supplementary Table S9. Correlation between the variation (**Δ**) of biomarker levels of the healthy controls group (T30-T0)

**Control Group (n=15)**						
**Δ (T30-T0)**	α-Syn, plasma	α-Syn, saliva	IL-6, plasma	IL-6, saliva	IL1-β, plasma	IL1-β, saliva
α-Syn, plasma	-	-.018	-	-	-	-
α-Syn, saliva	-	-	-	-	-	-
IL-6, plasma	**-.518**	-.318	-	-.196	-	-

IL-6, saliva	.125	-.064	-	-	-	-
IL1-β, plasma	.371	.282	**-.643**	.139	-	-.025
IL1-β, saliva	**.586**	-.146	-.396	-.157	-	-

* Spearman's correlation coefficient (rho). Significant (p<0.05) rho values denoted in bold. Abbreviations: n, number of participants; Δ, variation between baseline and follow-up; T0, baseline; T30, follow-up; α-Syn, alpha-synuclein; IL-6, interleukin 6; IL1-β, interleukin 1-β.

Supplementary Table S10. Correlation between biomarker levels of the overall sample at baseline (T0) and at 30-days follow-up (T30)

**Overall (n=26)**						
	α-Syn, plasma T30	α-Syn, saliva T30	IL-6, plasma T30	IL-6, saliva T30	IL1-β, plasma T30	IL1-β, saliva T30
α-Syn, plasma T0	**.902**	-.330	-.088	.151	-.032	.030
α-Syn, saliva T0	-.210	.127	.218	**-.492**	.653	.382
IL-6, plasma T0	.103	-.279	**.809**	.405	.056	.355
IL-6, saliva T0	.104	**-.562**	.095	**.854**	-.453	**-.478**
IL1-β, plasma T0	-.306	.065	.292	-.465	**.715**	.358
IL1-β, saliva T0	-.169	.344	.227	**-.675**	.385	**.775**

* Spearman's correlation coefficient (rho). Significant (p<0.05) rho values denoted in bold. Abbreviations: n, number of participants; Δ, variation between baseline and follow-up; T0, baseline; T30, follow-up; α-Syn, alpha-synuclein; IL-6, interleukin 6; IL1-β, interleukin 1-β.

Supplementary Table S11. Correlation between biomarker levels of the periodontitis group at baseline (T0) and at 30-days follow-up (T30)

**PG (n=11)**						
	α-Syn, plasma T30	α-Syn, saliva T30	IL-6, plasma T30	IL-6, saliva T30	IL1-β, plasma T30	IL1-β, saliva T30
α-Syn, plasma T0	**.782**	**-.673**	.128	.245	.218	-.282
α-Syn, saliva T0	.164	-.436	.556	-.264	.300	.227
IL-6, plasma T0	-.009	.155	.460	-.173	.055	.500
IL-6, saliva T0	.091	-.318	-.451	**.809**	-.227	**-.664**
IL1-β, plasma T0	.227	-.536	.588	-.200	.600	.173
IL1-β, saliva T0	-.282	.309	.451	**-.764**	-.055	**.755**

* Spearman's correlation coefficient (rho). Significant (p<0.05) rho values denoted in bold. Abbreviations: n, number of participants; Δ, variation between baseline and follow-up; α-Syn, alpha-synuclein; IL-6, interleukin 6; IL1-β, interleukin 1-β.

Supplementary Table S12. Correlation between biomarker levels of the control group at baseline (T0) and at 30-days follow-up (T30)

**HC (n=15)**						
	α-Syn, plasma T30	α-Syn, saliva T30	IL-6, plasma T30	IL-6, saliva T30	IL1-β, plasma T30	IL1-β, saliva T30
α-Syn, plasma T0	**.921**	-.075	-.193	.186	-.318	.154

α-Syn, saliva T0	-.289	**.636**	-.036	**-.729**	.800	.450
IL-6, plasma T0	.257	-.450	**.582**	.654	-.254	.011
IL-6, saliva T0	.143	**-.657**	-.061	**.857**	-.604	**-.518**
IL1-β, plasma T0	**-.581**	.533	.111	-.702	**.844**	.499
IL1-β, saliva T0	.054	.264	.282	**-.532**	.607*	**.811**

* Spearman's correlation coefficient (rho). Significant ($p<0.05$) rho values denoted in bold. Abbreviations: n, number of participants; T0, baseline; T30, follow-up; α-Syn, alpha-synuclein; IL-6, interleukin 6; IL1-β, interleukin 1-β.

Supplementary Table S13. STROBE Statement—Checklist of items that should be included in reports of ***case-control studies***

	Item No	Recommendation	Page
**Title and abstract**	1	(*a*) Indicate the study's design with a commonly used term in the title or the abstract	1
		(*b*) Provide in the abstract an informative and balanced summary of what was done and what was found	1
**Introduction**			
Background/rationale	2	Explain the scientific background and rationale for the investigation being reported	1-2
Objectives	3	State specific objectives, including any prespecified hypotheses	1-2
**Methods**			
Study design	4	Present key elements of study design early in the paper	8-10
Setting	5	Describe the setting, locations, and relevant dates, including periods of recruitment, exposure, follow-up, and data collection	8-10
Participants	6	(*a*) Give the eligibility criteria, and the sources and methods of selection of participants	8-10
Variables	7	Clearly define all outcomes, exposures, predictors, potential confounders, and effect modifiers. Give diagnostic criteria, if applicable	8-10
Data sources/ measurement	8*	For each variable of interest, give sources of data and details of methods of assessment (measurement). Describe comparability of assessment methods if there is more than one group	8-10
Bias	9	Describe any efforts to address potential sources of bias	8-10
Study size	10	Explain how the study size was arrived at	8-10
Quantitative variables	11	Explain how quantitative variables were handled in the analyses. If applicable, describe which groupings were chosen and why	8-10
Statistical methods	12	(*a*) Describe all statistical methods, including those used to control for confounding	8-10
		(*b*) Describe any methods used to examine subgroups and interactions	8-10
		(*c*) Explain how missing data were addressed	NA
		(*d*) If applicable, describe analytical methods taking account of sampling strategy	NA
		(*e*) Describe any sensitivity analyses	NA
**Results**			
Participants	13*	(a) Report numbers of individuals at each stage of study—eg numbers potentially eligible, examined for eligibility,	2-7

		confirmed eligible, included in the study, completing follow-up, and analysed	
		(b) Give reasons for non-participation at each stage	2-7
		(c) Consider use of a flow diagram	2-7
Descriptive data	14*	(a) Give characteristics of study participants (eg demographic, clinical, social) and information on exposures and potential confounders	2-7
		(b) Indicate number of participants with missing data for each variable of interest	NA
Outcome data	15*	Report numbers of outcome events or summary measures	2-7
Main results	16	(*a*) Give unadjusted estimates and, if applicable, confounder-adjusted estimates and their precision (eg, 95% confidence interval). Make clear which confounders were adjusted for and why they were included	2-7
		(*b*) Report category boundaries when continuous variables were categorized	2-7
		(*c*) If relevant, consider translating estimates of relative risk into absolute risk for a meaningful time period	2-7
Other analyses	17	Report other analyses done—eg analyses of subgroups and interactions, and sensitivity analyses	2-7
**Discussion**			
Key results	18	Summarise key results with reference to study objectives	7-8
Limitations	19	Discuss limitations of the study, taking into account sources of potential bias or imprecision. Discuss both direction and magnitude of any potential bias	7-8
Interpretation	20	Give a cautious overall interpretation of results considering objectives, limitations, multiplicity of analyses, results from similar studies, and other relevant evidence	7-8
Generalisability	21	Discuss the generalisability (external validity) of the study results	N/A
**Other information**			
Funding	22	Give the source of funding and the role of the funders for the present study and, if applicable, for the original study on which the present article is based	11
